# Bacterial Production, Characterization and Protein Modeling of a Novel Monofuctional Isoform of FAD Synthase in Humans: An Emergency Protein?

**DOI:** 10.3390/molecules23010116

**Published:** 2018-01-06

**Authors:** Piero Leone, Michele Galluccio, Alberto Barbiroli, Ivano Eberini, Maria Tolomeo, Flavia Vrenna, Elisabetta Gianazza, Stefania Iametti, Francesco Bonomi, Cesare Indiveri, Maria Barile

**Affiliations:** 1 Department of Bioscience, Biotechnology and Biopharmaceutics, University of Bari, via Orabona, 4, I-70126 Bari, Italy; pieroleone87@gmail.com (P.L.); maria.tolomeo89@gmail.com (M.T.); 2 Department of Biology, Ecology and Earth Science (DiBEST), Unit of Biochemistry and Molecular Biotechnology, University of Calabria, Via P. Bucci 4c, I-87036 Arcavacata di Rende, Italy; michele.galluccio@unical.it (M.G.); flaviavrenna@libero.it (F.V.); cesare.indiveri@unical.it (C.I.); 3 Dipartimento di Scienze per gli Alimenti, la Nutrizione e l’Ambiente (DeFENS), Università degli Studi di Milano, via G. Celoria 2, I-20133 Milano, Italy; alberto.barbiroli@unimi.it (A.B.); stefania.iametti@unimi.it (S.I.); francesco.bonomi@unimi.it (F.B.); 4 Gruppo di Studio per la Proteomica e la Struttura di Proteine, Dipartimento di Scienze Farmacologiche e Biomolecolari (DiSFeB), Università degli Studi di Milano, via Balzaretti 9, I-20133 Milano, Italy; ivano.eberini@icloud.com (I.E.); elisabetta.gianazza@unimi.it (E.G.); 5 Institute of Biomembranes, Bioenergetics and Molecular Biotechnology (IBIOM)—CNR, Via Giovanni Amendola 165/A-70126 Bari, Italy

**Keywords:** flavin, riboflavin, flavoprotein, FAD synthase, protein modeling, over-expression, *E. coli*

## Abstract

FAD synthase (FADS, EC 2.7.7.2) is the last essential enzyme involved in the pathway of biosynthesis of Flavin cofactors starting from Riboflavin (Rf). Alternative splicing of the human FLAD1 gene generates different isoforms of the enzyme FAD synthase. Besides the well characterized isoform 1 and 2, other FADS isoforms with different catalytic domains have been detected, which are splice variants. We report the characterization of one of these novel isoforms, a 320 amino acid protein, consisting of the sole C-terminal 3′-phosphoadenosine 5′-phosphosulfate (PAPS) reductase domain (named FADS6). This isoform has been previously detected in Riboflavin-Responsive (RR-MADD) and Non-responsive Multiple Acyl-CoA Dehydrogenase Deficiency (MADD) patients with frameshift mutations of FLAD1 gene. To functionally characterize the hFADS6, it has been over-expressed in *Escherichia coli* and purified with a yield of 25 mg·L^−1^ of cell culture. The protein has a monomeric form, it binds FAD and is able to catalyze FAD synthesis (k_cat_ about 2.8 min^−1^), as well as FAD pyrophosphorolysis in a strictly Mg^2+^-dependent manner. The synthesis of FAD is inhibited by HgCl_2_. The enzyme lacks the ability to hydrolyze FAD. It behaves similarly to PAPS. Combining threading and ab-initio strategy a 3D structural model for such isoform has been built. The relevance to human physio-pathology of this FADS isoform is discussed.

## 1. Introduction

Riboflavin, or vitamin B2, is the precursor of flavin mononucleotide (FMN) and flavin adenine dinucleotide (FAD), which are essential cofactors of hundreds of oxidoreductases (EC1) but also of other flavoproteins and flavoenzymes which are not oxidoreductases. These enzymes are involved in cellular metabolism and in several regulatory pathways, such as antioxidant defense, protein folding, chromatin remodeling and apoptosis [1,2,3,4].

These flavoenzymes are mainly located in the cellular organelles [5], where they ensure the functionality of the mitochondrial respiratory chain and of the Krebs cycle, and the metabolism of fatty acids, of some amino acids, of choline and betaine [6,7,8], and the synthesis of protoporphyrin [9]. Flavoenzymes are also implicated in the tetrahydrofolate-dependent one-carbon metabolism, as well as in the metabolism of vitamins B6, B9, and B12 [10,11,12,13].

Whereas bacteria are able to synthesize and export riboflavin [14,15], humans must obtain it as an essential nutrient via intestinal absorption from food or from the intestinal microbiota [16,17]. Riboflavin is transported into the bloodstream and taken up by target cells by the three human riboflavin transporters (RFVTs) recently identified as members of the solute carrier family SLC52A [10]. Mutations in RFVTs are causative of a rare neurodegenerative disorder named Brown-Vialetto-Van Laere syndrome and of a transient infantile form of RR-MADD [18,19,20].

Once in the cell, riboflavin is converted to FMN by riboflavin kinase (RFK, EC2.7.1.26) and FMN is converted, in turn, to FAD by a distinct enzyme, namely FMN:ATP adenylyl transferase, commonly known as FAD synthase (FADS, EC 2.7.7.2) or FAD synthetase [21,22]. Differently from mammals, prokaryotes synthesize FAD using a single bi-functional enzyme, which presents a C-terminal riboflavin kinase activity and a N-terminal FMN-adenylyl transferase (FMNAT) activity [23,24].

**Figure 1:**
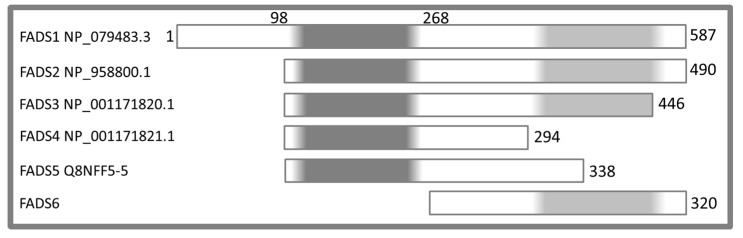
Schematic representation of the human FAD synthase protein isoforms. The protein length and the enzymatic activities of six human FAD synthase isoforms are reported. Molybdopterin binding-like domain, responsible of FAD hydrolysis, is indicated in dark grey (N-terminus); phosphoadenylyl sulfate reductase domain (PAPS), competent in FAD synthesis and in pyrophosphorolysis, is highlighted in light grey (C-terminus). hFADS1, hFADS2 and hFADS3 possess both catalytic domains; hFADS4 and hFADS5 present the sole molybdopterin binding domain; on the contrary, hFADS6 has the sole PAPS reductase domain. Numbers indicate amino acid positions in the resulting protein sequence of the FADS1 isoform.

In humans, distinct isoforms of FADS are the products of the FLAD1 gene (MIM: 610595), which is alternatively spliced in different transcript variants (Figure 1) that can be visualized by the UCSC (University of California Santa Cruz) Genome Browser. Two FADS isoforms deriving from the translation of seven exon long mRNAs have been characterized in detail [22,25,26]. The longest protein, namely FADS1, has a predicted mitochondrial targeting sequence (MTS), and a mitochondrial localization [27]. A slightly shorter isoform derives from interruption of exon 1 by an additional intron. This leads to usage of a downstream ATG coding Met98 of the longer transcript, resulting in FADS2 (Figure 1), with cytosolic localization; this 490 residues long enzyme was proposed to be a component of the FAD delivery machinery [25].

Both FADS isoforms contain a N-terminal molybdopterin binding (MPTb) domain, which has been shown to have FAD hydrolase activity [28], and a C-terminal 3-phosphoadenosine 5-phosphosulfate (PAPS) reductase domain, which per se performs the FAD synthase activity [29].

The expected central role of FADS in human muscle bioenergetics has been recently proved by the occurrence of certain riboflavin-responsive and non-responsive cases of Multiple Acyl CoA dehydrogenase deficiency (MADD, OMIM 231680) and/or of multiple respiratory-chain deficiency caused by mutations in FLAD1 [30,31]. The affected individuals show a severe metabolic myopathy, resembling what is seen in individuals with Electron Transfer Flavoprotein Dehydrogenase (ETFDH) defects [31,32,33]. Interestingly, the residual FADS activity detectable in these patients with homozygous frameshift variants in the MPTb domain led to the discovery of novel transcript isoforms, whose existence was validated by transcriptomic analysis. Theoretically, in humans, novel FADS isoforms, starting at Met268 (Figure 1) and at Met355, with respect to isoform 1, could be translated from the novel variant FLAD1 transcripts.

Since FLAD1 gene is the sole gene coding for FADS, these novel mono-functional isoforms, lacking of the MPTb domain, i.e., containing only the PAPS domain, might ensure cytosolic FAD synthesis and explain why affected individuals with biallelic FLAD1 frameshift variants still harbor substantial FADS activity and thus survive.

Even if the existence of these protein products was detected in MADD patient fibroblasts [30], the actual existence, the identity, and the functional and structural features of these isoforms are still a matter of investigation.

In the investigation accounted for in this report, we have over-produced in *E. coli* and purified with a yield sufficient to allow functional characterization and structure/function relationships studies the novel putative natural isoform starting at Met268 and named hFADS6. This isoform is able to perform both Mg^2+^-dependent FAD synthesis and the reverse reaction (i.e., FAD pyrophosphorolysis), but not FAD hydrolysis, the latter finding correlating with the lack of the molybdopterin binding domain in the novel variant (Figure 1).

## 2. Results

### 2.1. Cloning, Expression and Purification of the hFADS6 Isoform

**Figure 2:**
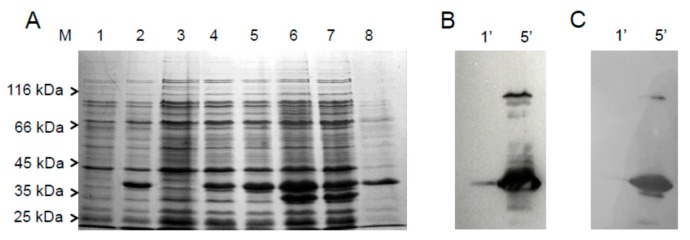
Over-expression and identification of recombinant hFADS6. (**A**) Proteins were separated by SDS–PAGE (Sodium Dodecylsulfate-Polyacrylamide Gel Electrophoresis) on 12% T polyacrylamide gel and stained with Coomassie Blue. Lane M: molecular mass markers: beta-galactosidase (116 kDa), bovine serum albumin (BSA) (66 kDa), ovalbumin (45 kDa), lactate dehydrogenase (35 kDa), restriction endonuclease Bsp98I (25 kDa); lane 1, non-induced cell lysate after 2 h of growth (6 µg); lane 2, cell lysate after 2 h of induction (8 µg); lane 3, soluble fraction of non-induced cell lysate after 2 h of growth (10 µg); lanes 4–6, soluble fractions of cell lysate after 2 h, 4 h and 8 h of 0.1 mM isopropyl β-d-1-thiogalactopyranoside (IPTG) induction at 28 °C, (12, 14 and 15 µg) respectively; lane 7, soluble fractions of cell lysate after 8 h of 0.1 mM IPTG induction at 20 °C (13 µg); lane 8, insoluble fraction of cell lysate after 4 h of 0.1 mM IPTG induction at 28 °C (7 µg); (**B**) Immunoblotting of hFADS6: lanes 1′ and 5′, immunodetection on the same protein fractions as in (**A**) with anti-FADS antiserum (1:2500); (**C**) lanes 1′ and 5′, immunodetection on the same protein fractions as in (**A**) with anti-His antibody (1:40,000). The molecular mass markers in (**B**,**C**) were as in (**A**).

The cDNA coding for human FADS isoform 6 (hFADS-6) was cloned between *Eco*RI and *Xho*I restriction sites of the pH6EX3 expression vector, generating a fusion peptide with a N-terminal 6-His-tag, whose theoretical molecular mass is 38.266 kDa. To optimize the heterologous production of the protein, several *E. coli* strains were screened for transformation, i.e., BL21(DE3)pLysS, JM109, RosettaGami2(DE3)pLysS, Rosetta(DE3)pLysS. *E. coli* Rosetta(DE3)pLysS strain, that is supplemented with transfer RNA (tRNA)-specific for rare codons, emerged as the best choice in terms of production of the recombinant protein (Figure 2).

Indeed, a band at about 36.0 kDa was present in the induced cell lysate after 2 h induction (Figure 2A lane 2), which was absent in non-induced cell lysate and supernatant (Figure 2A lanes 1 and 3, respectively). Different growth conditions and Isopropyl β-d-1-thiogalactopyranoside (IPTG) concentrations were tested to improve expression; IPTG concentration from 0.1 mM to 1 mM did not influence protein expression (not shown). The cell lysates were separated in a soluble and insoluble cell fraction as described in Materials and Methods. The amount of expressed protein recovered in the soluble fraction increased with time of growth (Figure 2A lanes 4–6). However, after 4 h, which was the best condition (Figure 2A, lane 5), protein degradation was also observed (Figure 2A lane 6), which was not prevented by lowering the temperature to 20 °C (Figure 2A, lane 7). A lower amount of protein was recovered in the cell pellet after 4 h induction (Figure 2A, lane 8), accounting for about 30% of the protein in the soluble fraction. The protein was identified by immunoblot performed with both the in-house produced anti-FADSs antibody (Figure 2B lane 1′ and 5′) and the anti-His antibody as a control (Figure 2C, lanes 1′ and 5′). In both cases a strong immunoreaction was observed in the induced cell lysate, which was virtually absent in the non-induced lysates. The faint immunoreaction at higher molecular mass might be due to aggregation of a very small fraction of the protein.

**Figure 3:**
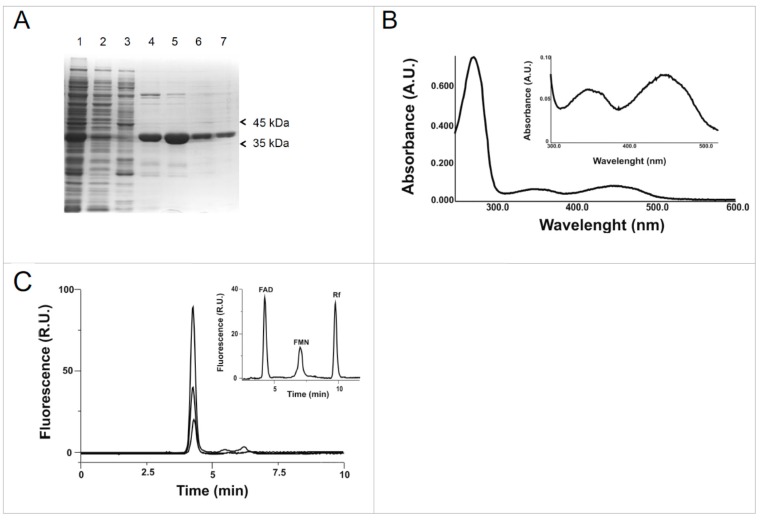
Purification of 6His-hFADS6. (**A**) Protein fractions obtained by Ni^2+^-chelating chromatography were separated by SDS–PAGE on 12% T polyacrylamide gel and stained with Coomassie Blue. Lane 1, soluble fraction of IPTG-induced cell lysate (30 μg); lane 2, first flow-through fraction (10 μg); lane 3, proteins eluted with 50 mM imidazole (8 μg); lane 4, first fraction of proteins eluted with 100 mM imidazole (9 μg); lane 5, second fraction of proteins eluted with 100 mM imidazole (10 μg); lane 6, first fraction of proteins eluted with 250 mM imidazole (5 μg); lane 7, second fraction of proteins eluted with 250 mM imidazole (4 μg); (**B**) Absorbance spectrum of the protein purified to homogeneity (Panel A, lane 7). The spectrum of hFADS6 (11.5 µM) was recorded in 40 mM Hepes/Na, 5 mM β-mercaptoethanol, pH 7.4. In the inset, a zoom in the visible region; (**C**) HPLC analysis of acid-extractable flavin cofactor. The purified protein (15.4 µg in 100 µL, 4 µM) was treated with 10% perchloric acid. The supernatant was neutralized and aliquots (10 and 20 µL) were analyzed for flavin content by HPLC. In the inset, chromatographic peaks of standard aqueous FAD (40 pmol), FMN (10 pmol) and riboflavin (10 pmol) solutions are shown for comparison.

To purify the protein of interest, the soluble fraction, corresponding to lane 5 of Figure 2 and to lane 1 of Figure 3, was applied on a Ni^2+^ chelating column and eluted as described in Materials and Methods. The column was then washed with 50 mM imidazole (Figure 3A lane 3); the elution of the 6His-hFADS6 was started with 100 mM imidazole (Figure 3A lanes 4 and 5) and was completed by increasing the imidazole concentration to 250 mM. The purified protein resulted in an apparently homogenous band (Figure 3A, lanes 6 and 7). The apparent molecular mass on SDS-PAGE was about 36 kDa, very close to the theoretical molecular mass of the 6His-hFADS6. The protein yield was about 25 mg·L^−1^ of cell culture. 

Spectrophotometric analysis of purified fraction 5 (Figure 3B) as well as of fractions 4, 6 and 7 (not shown) showed a typical flavoprotein absorbance spectrum, with a main peak at 274 nm and two minor peaks at 350 and 450 nm (Figure 3B) indicating that this novel purified isoform, as the previously characterized isoform 2, tightly binds flavins [25]. High performance liquid chromatography (HPLC) analysis of the supernatant obtained by acidic treatment of purified hFADS6 demonstrates that the bound cofactor is FAD (Figure 3C). The molecular ratio of FAD/monomer as determined by optical properties as well as by HPLC (see Materials and Methods) gave a value of 0.60 ± 0.015.

### 2.2. Molecular Characterization of hFADS6

**Figure 4:**
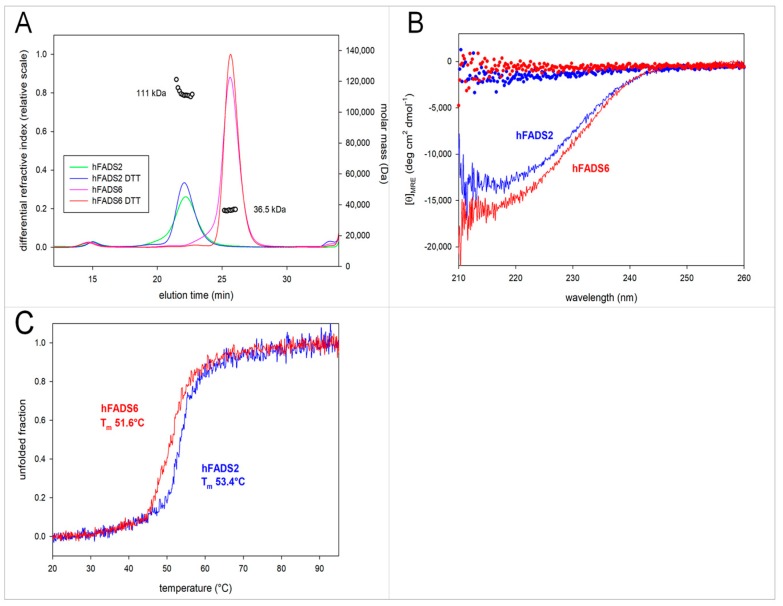
Characterization of the recombinant protein. (**A**) Tandem HPLC-GPC/LS profiles of 6-His-hFADS6 and 6-His-hFADS2. Proteins were separated on a Superose 12 column, connected in series to a dual wavelength detector, a refractive index detector, and a Dawn Heleos MALS system. The column was run in 40 mM Hepes/Na, 150 mM NaCl, pH 7.4, containing 1 mM DTT when indicated. Empty circles above individual protein peaks indicate the molecular mass calculated from MALS data; (**B**) Far-UV circular dichroism spectra of 6-His-hFADS6 and 6-His-hFADS2 in the absence (full lines) and in the presence (dotted lines) of 6 M urea. Spectra were recorded at 20 °C in 1-mm path length cells, on 0.2 mg·mL^−1^ protein solutions in 40 mM Hepes/Na, 150 mM NaCL, 1 mM DTT, pH 7.4; (**C**) Temperature ramp experiments were performed on 0.2 mg·mL^−1^ protein solutions in 40 mM Hepes/Na, 150 mM NaCl, 1 mM DTT, pH 7.4 by increasing the temperature from 20 °C to 95 °C at a heating rate of 1 °C min while continuously monitoring the loss of CD signal at 220 nm. Both FADS isoforms show a cooperative folding/unfolding transition. Data were processed to calculate the midpoint temperature (T_m_) for the native/denatured transition (53.4 °C for hFADS2 vs. 51.6 °C for hFADS6).

The aggregation state of hFADS6 and of hFADS2 was assessed by size exclusion chromatography and hydrodynamic radius measurement performed by tandem HPLC-GPC/LS. As shown in Figure 4A, hFADS6 migrated in HPLC-GPC as a single peak with an elution volume of 13 mL, much higher than what observed for hFADS2 (elution volume, 11 mL). The addition of 1 mM DTT to the elution buffer did not alter the chromatographic behavior of either protein, if not for improving the sharpness of individual peaks, likely because of the dissociation of minor amounts of disulfide linked aggregates. Online MALS (Multi-Angle Light Scattering) measurements performed on the peaks obtained in the presence of DTT gave molecular masses of 36.5 ± 5 kDa for hFADS6 and 111 ± 17 kDa for hFADS2, confirming that the hFADS6 and the hFADS2 had a monomeric and dimeric nature, respectively. 

hFADS6 has a CD signal originating from alpha-helical regions in secondary structure, and its CD spectrum nearly overlaps that of hFADS2 (Figure 4B), if not for minor differences in signal intensity, that could be related to the different length of the two isoforms. Within the limitations due to incomplete coverage of the far ultra-violet (UV) region and to the approximations inherent to these models, the alpha helical content in hFADS6 was estimated at 39%, comparable to 32% in hFADS2. The presence and thermal stability of secondary structure elements was assessed on either protein through temperature ramp experiments carried out by progressively heating protein solutions from 20 °C to 95 °C at 1 °C·min^−1^. In these experiments, both proteins gave a well-defined transition between the structured and the denatured state, confirming that even the shorter protein construct in hFADS6 has a defined structure. Tm values for the transition to denatured state in hFADS6 was 51.6 °C, slightly lower than the Tm measured for hFADS2 (53.4 °C). Whether the lower thermal stability of hFADS6 with respect to hFADS2 depends on the absence of structure-stabilizing features in the shorter isoform or on its monomeric state remains to be ascertained. 

### 2.3. Functional Characterization of hFADS6 Recombinant Protein

**Figure 5:**
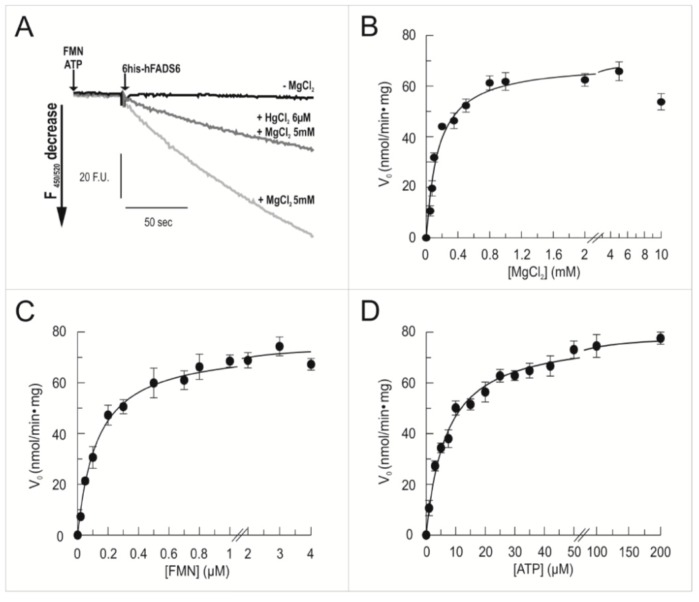
Fluorimetric evidence of FAD synthesis. (**A**) FAD synthesis was followed in 2 mL of 50 mM Tris/HCl, pH 7.5, containing 2 µM FMN and 100 µM ATP, at 37 °C, in the absence of MgCl_2_ (black line) and in the presence of 5 mM MgCl_2_ (light grey) or in the presence of 5 mM of MgCl_2_ plus 6 µM HgCl_2_ (dark grey). The FAD synthesis reaction was started by the addition of 6His-hFADS6 (0.11 µM) and measured by the initial rate of fluorescence decrease (λ excitation = 450 nm, λ emission = 520 nm); (**B**) MgCl_2_ concentration dependence. FAD synthesis rate, catalyzed by purified 6His-hFADS6 (0.13 µM), was measured fluorimetrically at 37 °C in 2 mL of 50 mM Tris/HCl pH 7.5, in the presence of 100 µM ATP, 2 µM FMN, and of the given MgCl_2_ concentrations; (**C**) FMN concentration dependence. FAD synthesis rate, catalyzed by purified 6-His–hFADS6 (0.13 µM) was measured fluorimetrically at 37 °C in 2 mL of 50 mM Tris/HCl pH 7.5, in the presence of 5 mM MgCl_2_, 100 µM ATP, and of the given FMN concentrations; (**D**) ATP concentration dependence. FAD synthesis rate, catalyzed by purified 6-His–hFADS6 (0.13 µM), was measured fluorimetrically at 37 °C in 2 mL of 50 mM Tris/HCl pH 7.5, in the presence of 5 mM MgCl_2_, 2 µM FMN and of the given ATP concentrations.

To ascertain if the over-expressed recombinant hFADS6 protein was able to synthesize FAD, the purified protein was incubated with the substrates, in the absence or presence of Mg^2+^, which is specifically required by other human FADS isoforms [21,22]. The FAD synthesis reaction (Figure 5A) was started by the addition of 0.13 µM purified hFADS6 to ATP (100 µM) and FMN (2 µM). An evident fluorescence decrease (λ excitation at 450 nm, λ emission at 520 nm) was observed, indicating the conversion of the highly fluorescent FMN into the less fluorescent FAD. The initial rate of FAD synthesis in the cuvette, calculated as described in Materials and Methods, was about 0.49 nmol·min^−1^ (corresponding to 2.3 min^−1^ in this preparation, 2.7 ± 0.13 min^−1^ as the mean of three different protein preparations.) As expected for FADSs, in the absence of Mg^2+^, nearly no fluorescence decrease was observed [21,22].

A relevant feature of hFADS2 is the presence of redox reactive cysteines, some of which implicated in the catalytic activity [26]. To directly evaluate if some Cys residues of hFADS6 play a role in enzyme function, the effect of HgCl_2_ was tested. Addition of HgCl_2_ inhibited the FAD synthesis reaction, in agreement with previous data described for the truncated form of FADS missing the N-terminal domain [26]. This inhibitory effect corroborates the proposal that free Cys residues located in the PAPS domain participate in the catalytic cycle of the hFADSs.

Kinetic analyses of FAD synthesis as function of MgCl_2_ (Figure 5B), FMN (Figure 5C) or adenosine triphosphate (ATP) (Figure 5D) concentration were performed. A hyperbolic dependence was found for FAD synthetic rate on Mg^2+^ concentration, with 50% of maximum activity (Mg^2+^_50_) at 0.15 mM ± 0.02 mM, in line with hFADS2 values [25]. Data fitting according to the Michaelis–Menten equation using the Grafit program (see Material and methods) gave apparent values of: K_m_fmn 0.13 ± 0.01 µM and K_m_atp 6.91 ± 0.51 µM (data from 3 independent experiments). Overlapping results were obtained by interpolating the same data in the Lineweaver-Burk equation using the Grafit program (not shown). These experiments gave k_cat_ values of 2.8 and 3.0 min^−1^, respectively. In three different experiments a mean value of 2.9 ± 0.05 min^−1^ was evaluated.

**Figure 6:**
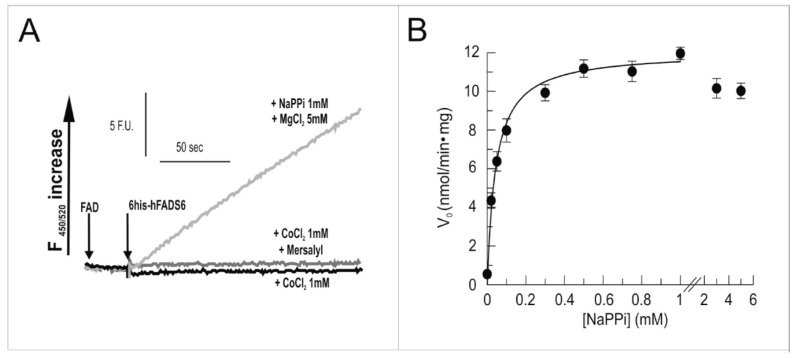
Fluorimetric evidence for FAD cleavage as catalyzed by 6-His–hFADS6. (**A**) Fluorimetric evidence of FAD cleavage. The reaction was measured in 50 mM Tris/HCl, pH 7.5, containing 0.5 µM FAD and 1 mM NaPPi, at 37 °C, in the presence of 5 mM MgCl_2_ (light grey). The FAD cleavage reaction (i.e., pyrophosporolysis) was started by the addition of the 6-His-hFADS6 protein (0.13 µM) and was measured by the initial rate of fluorescence increase (λ excitation = 450 nm, λ emission = 520 nm). FAD hydrolysis was measured under the same conditions, if not for omitting NaPPi and MgCl_2_, and in the presence of either 1 mM CoCl_2_ (black line) or 1 mM CoCl_2_ plus 0.5 mM Mersalyl (dark grey); (**B**) NaPPi concentration dependence. FAD cleavage (i.e., pyrophosporolysis) rate, catalyzed by purified 6-His–hFADS6 (0.13 µM), was measured fluorimetrically at 37 °C in 2 mL of 50 mM Tris/HCl pH 7.5, in the presence of 5 mM MgCl_2_, 0.5 µM FAD, and of the given NaPPi concentrations.

The reverse reaction, i.e., pyrophosphorolysis, was also tested, as well as the ability to cleave FAD by hydrolysis, as previously reported for the bi-functional isoform 2 [28]. As expected on the basis of the lack of the MPTb domain, isoform 6 of the enzyme was able to catalyze the pyrophosphorolysis, but not the hydrolysis (Figure 6A); not even the presence of Co^2+^ could promote the ability of hFADS6 to catalyze hydrolysis, at a difference from the bi-functional variant. A control was also made by using the -SH reagent mersalyl. Mersalyl induces FAD hydrolysis in the hFADS2 isoform, but was found inactive in this regard with the hFADS6 variant. The kinetics of the reverse reaction was measured as a function of NaPPi concentration (Figure 6B). A K_m_ppi of 0.042 ± 0.006 mM was calculated by interpolating the data in the Michaelis-Menten equation. The K_m_fad could not be measured due to instrumental limitations that do not allow measurements of K_m_ values lower than 0.1 μM as in the case hFADS6, similarly to hFADS2 [25].

### 2.4. 3D Model of hFADS6

**Figure 7:**
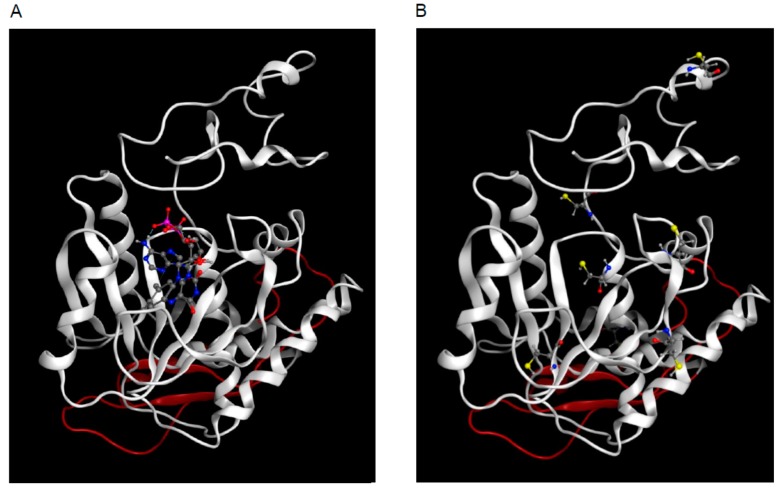
Three-dimensional (3D) model of hFADS6. Ribbon representation of hFADS6 modeled through Robetta as described in Materials and Methods. (**A**) FAD has been transferred in the active site from PDB ID: 3g6k and is represented in ball and stick. The PAPS reductase domain is colored in white, the N-terminus part of hFADS6 is colored in red. (**B**) Cys residues are represented in ball and stick and colored by elements. Element colors in (**A**,**B**): Sulfur, yellow; Nitrogen, blue; Oxygen, red; Phosphorus, pink; Carbon, grey.

Structural models of hFADS6 apo-protein were computed with three different programs, using homology and/or threading procedures. The C-terminus part of hFADS6 (PAPS domain) is homologous to already experimentally characterized proteins, with identity/similarity levels adequate for successfully producing homology models with an accurate architecture: for instance, query primary structure (aa 109–320) has a 36% similarity with *Saccharomyces cerevisiae* YDL045c FAD synthetase. Accordingly, the output from the three different modeling approaches was very similar when predicting by homology modeling the organization of the C-terminus part of hFADS6. On the contrary, the produced models largely diverged in the arrangement of the N-terminus part, for which no homologous templates can be identified. For this reason, Swiss Model, which is a software exclusively based on homology modeling, failed in predicting 3D structure of the hFADS6 N-terminus. Robetta or I-TASSER, which also implement ab initio modeling, produced dissimilar 3D structures for this part. The output provided by Robetta was selected as final apo-hFADS6 model, for its better geometrical features. FAD ligand was transferred, after superposition with the crystallographic structure of the *Candida glabrata* FMN Adenylyltransferase (PDB ID: 3g6k) for generating the holo-hFADS6 form (Figure 7, PAPS domain in white, N-terminus part in red). In more general terms, the predicted model for hFADS6 had an alpha-helical content of 37% (117 residues out of 320), close to that calculated from CD spectra (39%). The content of beta-strands cannot be determined by the limited coverage of our CD spectra, but it was estimated at 10% in the model (32 residues out of 320).

## 3. Discussion

The FADS6 isoform was very recently discovered, by transcriptome analysis, in patients harboring frameshift mutations of FLAD1 gene triggering RR-MADD. This FADS6 isoform can be anyway produced from the altered gene since it starts from Met268 whose codon is downstream the frameshift ([30], and Figure 1). However, it was not known if this isoform could display some functions related to FAD production. We have demonstrated in this work that this shorter isoform is able to synthesize FAD. Therefore, its importance, even though expressed in small amount, resides in human pathophysiology, that is, to synthesize sufficient amount of FAD, allowing survival of patients affected by disruptive mutations of the FLAD1 gene. hFADS6 harbors additional 61 N-terminus amino acid residues with respect to the artificial PAPS mutant. Hence, it could then be considered the natural counterpart of the artificial protein previously produced by recombinant DNA strategies [29]. Therefore, it was very important to ascertain the capacity of the novel hFADS6 isoform to synthesized FAD. FADS6 displayed FAD synthesizing activity, but not FAD hydrolase activity, which resides in the N-terminal domain of the full length FADS1 or 2 [28]. Moreover, FADS6 showed some similar features to the other previously characterized longer FADS isoforms, such as Mg^2+^ dependence and strong inhibition by Hg^2+^, confirming that some of the Cys residues highlighted in the model (Figure 7) play crucial roles in this PAPS domain [26]. The FAD synthesizing activity is about 70% with respect to that measured for hFADS2 [25]; however, the catalytic efficiency (k_cat_/K_m_) results higher than that of hFADS2 due to the lower Km values of hFADS6 for both FMN and ATP compared to those of hFADS2. The data are in agreement with those found for PAPS [29]. Concerning the Mg^2+^ binding, we expect a similar behavior as in *C. glabrata* FMN Adenylyltransferase, i.e., Mg^2+^ makes a bridge between bound ATP and the protein binding site. In fact, hFADS6 harbors two residues, D141 and D225, which are homologous to those responsible for Mg^2+^ binding in *C. glabrata* enzyme (D66 and D168) [34].

From a structural standpoint, it is noteworthy that the short and monomeric hFADS6 appears to be appropriately folded, with a thermal stability of its folding remarkably close to that of the much larger (and dimeric) hFADS2. The presence of DTT has effect neither on the aggregation state nor on the hydrodynamic radius of the two proteins, confirming the absence of structurally relevant disulfide bonds in the structure. 

In agreement with the high sequence identity between the two primary structures, the models for the 3D structure of the C-terminus part of hFADS6 and of the PAPS mutant previously produced through recombinant DNA are similar to one another (Figure 7, white structure), except for the N-terminal 61 amino acids of the hFADS6 (Figure 7, red structure). Besides the overall close similarity of the fold, even the positions of the Cys residues in the two structures mostly overlap [29]. Thanks to the availability of a number of templates with similarity with the target higher than 30%, the prediction of the general architecture of this part of hFADS6 can be considered accurate. Conversely, lack of adequate templates forces to rely on threading and ab initio modeling for the N-terminus part of hFADS6, accepting a lower confidence of the outcome. Indeed, the results provided by Robetta and I-TASSER for the N-terminus of the hFADS6 diverge for their overall orientation with respect to the PAPS domain. In neither case, however, the access to the catalytic site is hampered by the N-terminus part of the protein. Similarity of the 3D arrangement of these two isoforms in the C-terminus part is the structural basis for a conserved catalytic activity. Indeed, docking of FAD on hFADS6 (Figure 7A) led to similar results with respect to the PAPS [29]. 

Despite the hFADS6 synthase function overlaps that of hFADS2, clear structural differences were observed between the two proteins. Indeed, differently from the dimeric structure of hFADS2, hFADS6 had an unequivocal monomeric form. Since the major difference in hFADS6 with respect to hFADS2 is the lack of the molybdopterin binding domain, this difference could be the cause of the absence of the dimeric structure. In other words, in hFADS2, the molybdopterin binding domain should play some role in the interaction between the two monomers. Indeed, previous studies based on site-directed mutagenesis addressed the relationships between the molybdopterin binding and the PAPS domains [26]. Further conditioning of the structure determined by the redox state (SH/S-S balance) of the protein is likely to occur; a crucial role for some Cys residues in the PAPS domain of the hFADS2 was assessed by site-directed mutagenesis. This issue is under investigation for hFADS6 in comparison with the hFADS2 isoform.

The first evidence of the expression of hFADS6 isoform in human tissues was provided in 2016 [30]. However, a clear correlation on expression level in healthy and pathological conditions is still lacking. Another interesting question concerns the putative localization of this novel isoform. Since this hFADS6 lacks the mitochondrial targeting peptide [27] a sole cytosolic localization is expected. This point is particularly relevant for cellular bioenergetics. Indeed, the relative activity of mitochondrial vs. cytosolic isoforms of FADS might regulate the transport directionality of the mitochondrial FAD transporter [35]. Therefore, under the conditions in which frameshift mutations of FLAD1 gene alter the relationships among splice variants, this novel emergency protein becomes relevant and we expect that flavoprotein biogenesis in mitochondria is ensured by FAD import. Experiments are on the way to directly prove these assumptions in the frame of measuring the relative abundance of the natural hFADS6 in different tissues. 

In conclusion, FADS6 represents a previously neglected FADS isoform whose role was highlighted by the discovery of FADS gene natural mutations causative of RR-MADD [30]. Very importantly, this isoform may represent an emergency protein, which can be produced also in patients from an altered gene. In this respect, this protein represents a target for therapy intervention in patients harboring FADS defects, especially for those in which Rf therapy is unsuccessful.

## 4. Materials and Methods

### 4.1. Materials

All chemicals and monoclonal anti-polyhistidine-peroxidase antibody (A7058) were from Sigma-Aldrich (Saint Louis, MO, USA), if not otherwise specified. *E. coli* Rosetta(DE3)pLysS strain was purchased from Novagen (Madison, WI, USA). Restriction endonucleases and other cloning reagents were purchased from Fermentas (Burlington, ON, Canada); MegaMan Human Transcriptome Library was from Agilent Technologies (Santa Clara, CA, USA). Anti-hFADSs antibody (patent number WO2009107158 A1) was in-house produced.

### 4.2. Cloning of cDNA Coding for hFADS6

The 963 bp cDNA coding for the hFADS6 isoform [30] was amplified from the Mega Man Human Transcriptome Library with the forward and reverse primers 5′-CCG*GAATTC*TATGAAGGGACTATTCCAAAACCCAG-3′ and 5′-CCG*CTCGAG*CTACCCCTGCTGTCCTGGGAAGGGG-3′, containing the *Eco*RI and *Xho*I sites, respectively. The amplified cDNA was then cloned in the *Eco*RI/*Xho*I sites of the pH6EX3 expression vector. The resulting recombinant plasmid, defined as pH6EX3-hFADS6, encodes a 6His-tagged fusion protein corresponding to hFADS6 carrying the extra N-terminal sequence MSPIHHHHHHLVPRGSEASNS.

### 4.3. Expression of Recombinant hFADS6 Protein in E. coli

The expression host *E. coli* Rosetta(DE3)pLysS strain was transformed with the pH6EX3-hFADS6 construct by calcium chloride treatment. Selection of positive colonies was performed on LB-agar plates containing 34 µg·mL^−1^ chloramphenicol and 100 µg·mL^−1^ ampicillin. *E. coli* Rosetta(DE3)pLysS cells carrying the recombinant plasmid were inoculated in 10 mL of LB medium (1% Bacto Peptone, 0.5% Bacto Yeast extract, 1% NaCl, pH 7.0) supplemented with 100 µg·mL^−1^ ampicillin and 34 µg·mL^−1^ chloramphenicol, and cultured overnight at 37 °C with rotary shaking (160 rpm). A 5 mL aliquot of the cell culture was transferred to 0.5 L of fresh LB medium supplemented with 100 µg·mL^−1^ ampicillin and 34 µg·mL^−1^ chloramphenicol and grown at 37 °C to A_600_ equal to 0.6–0.8. Then, 0.4 mM IPTG was added to induce the expression of the recombinant protein, 6-His-hFADS6. Growth was continued for 2 to 8 h at 28 °C, or overnight at 20 °C. Bacteria were harvested by centrifugation at 3000×*g* for 10 min at 4 °C and the pellets stored at −20 °C. The bacterial pellet (about 3 g wet weight) was thawed on ice for 15 min and resuspended in 30 mL of start buffer (500 mM NaCl, 40 mM Hepes/Na, pH 7.4) supplemented with Protease Inhibitor Cocktail (P8849, Sigma-Aldrich, 1 mL/20 g of cells wet weight) and 0.5 mM Phenylmethylsulfonyl fluoride (PMSF). Cells were disrupted by mild sonication at 4 °C (one cycle of 10 min and one cycle of 5 min with 1 s Pulse ON and 1 s Pulse OFF, at 40 W) using a VCX130 PB sonifier (Branson, Danbury, CT, USA). The soluble and the insoluble cell fractions were separated by centrifugation of the cell lysate at 20,000×*g* for 30 min at 4 °C. The pellet (inclusion bodies and cell debris), containing the insoluble over-expressed proteins, was re-suspended in 15 mL of start buffer, aliquoted and used for SDS-PAGE analysis. The supernatant, containing the soluble over-expressed 6-His-hFADS6, was used for SDS-PAGE analysis, FADS activity assay, and further protein purification (see below).

### 4.4. Purification of Recombinant 6-His-hFADS6

A 40 mL aliquot of the soluble cell fraction, obtained as outlined above, was applied onto a Chelating Sepharose Fast Flow column (3.5 mL packed resin), previously treated with 200 mM NiSO_4_ according to the producer’s protocol and equilibrated with the start buffer. The column was first washed with 35 mL start buffer, then eluted with a step gradient of 50, 100, 250, and 500 mmol·L^−1^ imidazole in the same buffer. Prior to storing or further processing, fractions containing the purified recombinant protein were desalted by gel filtration on a PD10-column in 40 mM Hepes/Na, 5 mM β-mercaptoethanol, pH 7.4. These protein samples were stable for at least 30 days at 4 °C.

### 4.5. Protein Concentration and FAD/Protein Monomer Ratio Measurements

Protein concentration was measured with the method of Bradford, using BSA as standard [36]. In an alternative procedure, protein concentration of the purified 6-His-hFADS6 was estimated by absorbance spectra, which were recorded on an Ultrospec 3100 pro spectrophotometer (Amersham Biosciences, Piscataway, NJ, USA), essentially as in [25]. To this aim, the contribution of the bound FAD had to be subtracted from the A_280_ readings. Because A_280_ for both free FAD and hFADS6-bound FAD is 1.7-fold A_450_, the A_280_, actually due to the apo-protein, may be calculated from the Equation:

A_280_ apo-enzyme = A_280_ − (A_450_ × 1.7)

The protein concentration was then estimated by using ε_280_ (47.705 mM^−11^·cm^−1^, 1.247 mg/mL^−1^·cm^−1^), as calculated from the protein sequence by using the ExpasyProtParam tool (Swiss Institute of Bioinformatics, Lausanne, Switzerland). Measurements made by either the spectrophotometric or the Bradford method differed by no more than 7%. The FAD/protein monomer ratio (given as percent flavinylation, Fl%) could be estimated from the absorbance spectrum by considering:

Fl% = [(A_450_/A_280_apo-enzyme)/0.23] × 100

where 0.23 is the ε_450_ (FAD)/ε_280_ (apo-enzyme) ratio.

### 4.6. FAD Content as Assayed by HPLC Analysis

The purified 6-His–hFADS6 (15.4 µg in 100 µL, 4 µM) was treated with 10% perchloric acid to remove tightly but not covalently bound molecules. The supernatant was neutralized and aliquots were analyzed for flavin content by HPLC, as previously reported [25,37]. Standard aqueous of FAD, FMN and riboflavin were used to identify the protein-bound flavin cofactor.

### 4.7. Circular Dichroism Measurements

Circular dichroism (CD) spectra were recorded at 20 °C in 0.1 cm path length cells on 0.2 mg·mL^−1^ protein solutions in 40 mM Hepes/Na, 150 mM NaCl, 1 mM DTT, pH 7.4 by means of a J810 spectropolarimeter (Jasco Europe SrL, Cremella, Italy), and were analyzed by means of the J800 software (Jasco Europe SrL, Cremella, Italy). Similar conditions were used for temperature ramp experiments. Temperature was increased from 20 to 95 °C at a heating rate of 1 °C·min^−1^ by means of a computer-controlled Peltier-driven cell holder, while continuously monitoring the loss of CD signal at 220 nm. CD spectra were normalized in terms of Mean Residual Ellipticity ([Ө]_MRE_, [38]), and the alpha-helical content was estimated from the [Ө]_MRE_ at 222 nm according to Chen and Yang [39].

### 4.8. Molecular Parameters

The molecular size of the purified 6-His–hFADS6 was assessed by HPLC-GPC, using a Superose 12 column (10/300 GL, GE Healthcare, Milan Italy), fitted to a HPLC system (mod. 515) equipped with a tunable dual wavelength detector (Waters 2487, Milford, MA, USA) and a refractive index detector (Optilab^®^ T-rEX, Wyatt, Santa Barbara, CA, USA). Columns were run at 0.5 mL·min^−1^ in 40 mM Hepes/Na, 150 mM NaCl, pH 7.4, containing 1 mM DTT when indicated, and calibrated with suitable protein standards that encompassed the molecular size predicted for hFADS2. The hydrodynamic properties and the molar mass of the purified 6-His–hFADS6 were assessed by Multi Angle Light Scattering (MALS), using a Wyatt Dawn^®^ Heleos^®^ system connected in series to the aforementioned HPLC-GPC chromatographic system. Molar mass at different elution times was calculated by means of the Astra software (Wyatt, Santa Barbara, CA, USA), using a dn/dc value of 0.185.

### 4.9. Measurements of FAD Synthesis and FAD Pyrophosphorolysis Rate

The rate of FAD synthesis and FAD cleavage were measured as in [25], by exploiting the different fluorescence properties of FAD with respect to FMN. Fluorescence time courses (λ excitation at 450 nm; λ emission at 520 nm) were followed at 37 °C in a FP-3800 spectrofluorometer (Jasco, Easton, MD, USA). In each experiment, FAD and FMN fluorescence were calibrated by using standard solutions. The rate of FAD synthesis, expressed as nmol FAD min^−1^·(mg protein)^−1^, was calculated from the rate of fluorescence decrease, measured as the tangent to the initial part of the experimental curve, as described in detail in [25]. For activity measurements, purified protein fractions (5–10 µg, 0.13–0.26 nmol protein as monomer, unless otherwise indicated) were incubated at 37 °C, in 50 mM Tris/HCl, pH 7.5, containing 5 mM MgCl_2_, 2 µM FMN, 100 µM ATP, and additional reagents as appropriate.

The rate of FAD pyrophosphorolysis was measured in the presence of 1 mM NaPPi (sodium pyrophosphate) and 0.5 µM FAD as in [25]. The rate of FAD cleavage was expressed as nmol FAD min^−1^·(mg protein)^−1^, and was calculated from the rate of fluorescence increase, measured as the tangent to the initial part of the experimental curve. Under the same experimental conditions, but in the absence of NaPPi, the possible occurrence of FAD hydrolysis was investigated essentially as in [28,40], in the presence of 1 mM CoCl_2_. 

To fit the experimental data and to obtain estimates of the kinetic parameters, the Grafit software was used (v. 5.0.13, 2006, by R.J. Leatherbarrow, Erithacus Software, Wilmington House, UK).

### 4.10. Electrophoretic Analysis

Proteins were separated by SDS-PAGE on 12% T polyacrylamide gels, according to Laemmli [41]. Quantitative evaluation of Coomassie Blue-stained protein bands was carried out using the Chemidoc imaging system (Bio-Rad, Hercules, CA, USA) and the Quantity One software (Bio-Rad, Hercules, CA, USA), as described previously [42].

### 4.11. Immunoblotting

SDS-PAGE-separated proteins were electro-transferred onto a nitrocellulose membrane using a trans-blot semidry electrophoretic transfer cell (Eppendorf, Hamburg, D). The immobilized proteins were incubated 1 h either with a 2500-fold dilution of an in-house produced polyclonal antiserum against hFADSs or with a 12,000-fold dilution of a peroxidase-conjugated anti-polyhistidine antibody. The bound antibodies were visualized with the aid of a secondary anti-rabbit immunoglobulin antibody, conjugated with alkaline phosphatase (1:5000 dilution).

### 4.12. 3D Modeling of hFADS6

A three-dimensional model was obtained by submitting the target sequence to three homology and/or ab initio servers: Swiss Model (https://swissmodel.expasy.org), I-TASSER (https://zhanglab.ccmb.med.umich.edu/I-TASSER/) and Robetta (http://robetta.bakerlab.org) and by comparing their outputs. Swiss Model could only provide results for the C-terminal domain, whereas the threading/ab initio combined procedures implemented by the other two approaches produced almost complete models. Of the latter two, the model built by Robetta scored better in terms of protein geometry (Ramachandran plot, bond lengths, angles, dihedrals, rotamers, clashes, and contact energies), and was thus selected. FAD was transferred to it from one of the templates used by I-TASSER (namely, 3g6k); the resulting complex was then energy minimized with the Amber10:EHT forcefield and the reaction field electrostatics, by the Molecular Operating Environment v. 2016.0803.
